# A deep learning approach to real-time HIV outbreak detection using genetic data

**DOI:** 10.1371/journal.pcbi.1010598

**Published:** 2022-10-14

**Authors:** Michael D. Kupperman, Thomas Leitner, Ruian Ke

**Affiliations:** 1 Theoretical Biology and Biophysics (T-6), Los Alamos National Laboratory, Los Alamos, New Mexico, United States of America; 2 Department of Applied Mathematics, University of Washington, Seattle, Washington, United States of America; Temple University, UNITED STATES

## Abstract

Pathogen genomic sequence data are increasingly made available for epidemiological monitoring. A main interest is to identify and assess the potential of infectious disease outbreaks. While popular methods to analyze sequence data often involve phylogenetic tree inference, they are vulnerable to errors from recombination and impose a high computational cost, making it difficult to obtain real-time results when the number of sequences is in or above the thousands.

Here, we propose an alternative strategy to outbreak detection using genomic data based on deep learning methods developed for image classification. The key idea is to use a pairwise genetic distance matrix calculated from viral sequences as an image, and develop convolutional neutral network (CNN) models to classify areas of the images that show signatures of active outbreak, leading to identification of subsets of sequences taken from an active outbreak. We showed that our method is efficient in finding HIV-1 outbreaks with *R*_0_ ≥ 2.5, and overall a specificity exceeding 98% and sensitivity better than 92%. We validated our approach using data from HIV-1 CRF01 in Europe, containing both endemic sequences and a well-known dual outbreak in intravenous drug users. Our model accurately identified known outbreak sequences in the background of slower spreading HIV. Importantly, we detected both outbreaks early on, before they were over, implying that had this method been applied in real-time as data became available, one would have been able to intervene and possibly prevent the extent of these outbreaks. This approach is scalable to processing hundreds of thousands of sequences, making it useful for current and future real-time epidemiological investigations, including public health monitoring using large databases and especially for rapid outbreak identification.

## Author summary

The analysis of pathogen genomic data to analyze epidemics at scale is constrained by the computational cost associated with phylogenetic tree reconstruction. As a fast and efficient alternative, we employed convolutional neural networks to analyze evolutionary pairwise distance matrices as images to perform classifications of the current epidemiological situation of a growing public health sequence database. We used simulated data to train and test our model, and as validation we accurately mapped the start and end of two linked well-documented HIV-1 outbreaks in the backdrop of ongoing slower HIV spread. Thus, our new approach is efficient, accurate, scalable, and can analyze data in real time.

This is a *PLOS Computational Biology* Methods paper.

## Introduction

The human population is increasingly exposed to threats of infectious disease outbreaks due to population growth, increased frequency of traveling, changing patterns of land use etc. This is exemplified by the ever-growing HIV epidemic [1] as well as recent emerging outbreaks such as the SARS-CoV-2 pandemic. A key to outbreak control is early detection when the number of infected individuals is small and the disease spread is local. One growing resource for disease control is to utilize pathogen genomic sequence data to assess epidemiological conditions and threats.

Given sequence data, state-of-the-art, flexible phylogenetic methods have been developed for analysis of general evolutionary questions [2–4], applicable to pathogen evolution, as well as faster but less precise algorithms for large data [5]. More focused phylodynamic methods have also been developed for specific tasks, e.g., taking incidence data into account [6], including multiple evolutionary scales [7], inferring underlying transmission networks on several levels [8, 9], and using large next generation sequencing data [10]. Motivated by pathogen evolution, advanced methods for inference of past demographic history with population size dynamics and migration that can reconstruct outbreaks have also been developed, e.g., the modular framework of BEAST [11], which takes a Bayesian approach to account for uncertainties in the tree reconstruction. However, phylogenetic tree reconstruction, interpretation, and subsequent outbreak identification requires extensive expert knowledge, and thus typically can be reliably done only by highly trained scientists.

One popular alternative to phylogenetic inference is HIV-TRACE [12], which aims to identify clusters of connected components by applying a threshold distance to a genetic distance matrix for a set of sequences. HIV-TRACE has been used in scientific investigations [13–16], as well as in public health settings in the USA [17].

Here,we propose an alternative to phylogenetic reconstruction and HIV-TRACE. We based our approach on the pairwise distance matrix of a sample of genetic sequences, but used a deep learning approach to analyze the matrix (instead of using a distance threshold as in HIV-TRACE). Deep learning approaches, such as the convolutional neural network (CNN) [18], has been well developed and widely used for image identification over the past decade [19–21]. By using many parameters in a highly nonlinear model, a deep learning model can efficiently learn complex relationships within the data to form highly accurate predictions [22]. Our rationale here is that outbreaks would lead to distinctive signatures in the pairwise distance matrix (similar to its impact on the topology of the phylogenetic tree [23]). We leveraged the advance in deep learning models by treating the matrix as an image, and developed deep learning models to identify these signatures from the pairwise distance matrix and thus detect outbreaks from a sequence database. Using simulated data, we show that our CNN model can accurately identify viral sequences belonging to an outbreak. It performed better than HIV-TRACE. In addition, we show that our CNN models made accurate predictions against historical HIV sequence data with known epidemiological history, and that they can handle many thousands of sequences within a very short time frame using a laptop computer.

## Methods

### Overview of the framework

**Fig 1:**
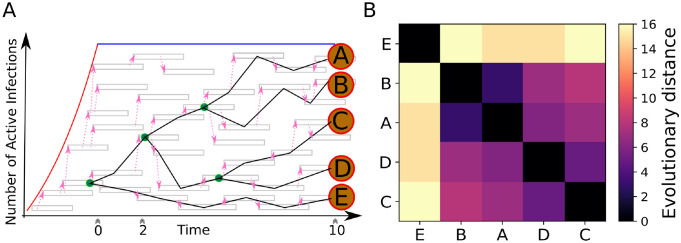
(A) Cartoon representation of the biphasic forward stochastic simulation. The red line indicates the exponential phase, the blue line indicates the constant population phase. Grey boxes represent an infected individual, pink arrows indicate transmission. The reconstructed transmission dendrogram is overlaid, green nodes are internal, orange (lettered) nodes indicate sampled infections. Triple chevrons indicate different possible sampling times (0, 2, 10 years). (B) The resulting image representation of the sorted pairwise distance matrix sampled at Year 10.

Here we describe the main workflow of our approach. We first developed a forward stochastic simulator of HIV transmission to generate synthetic datasets for training and testing of our CNN models. In this simulator, the number of infected individuals initially expand exponentially, and subsequently establish a constant population size (Fig 1A). The entire transmission history was recorded; *n* individual samples (*n* = 15, 20, 30, 40, or 50 in our model) were taken either during the exponentially increasing phase (labeled as ‘epidemic’) or the constant population phase (labeled as ‘endemic’). Nucleotide substitutions were then simulated on the transmission tree/genealogy of the *n* sampled individuals, and a *n* × *n* pairwise distance matrix was derived from the simulated substitutions on the transmission tree (Fig 1B). We repeated the stochastic simulation many times to derive a rich synthetic dataset (i.e. a collection of matrices with labels).

We then developed CNN models, trained and tested these CNN models using the synthetic dataset to identify matrices that are labeled as ‘epidemic’. These CNN models handle a small number of sequences (*n* = 15, 20, 30, 40, or 50) at a time; however, a pathogen database may contain tens of thousands or even hundreds of thousands of sequences. We thus developed a ‘sliding-window’ approach to utilize the CNN model to identify subsets of sequences that belong to an active outbreak, i.e. the ‘epidemic’ label, from a collection of a large number of sequences. More specifically, we first constructed a pairwise distance matrix for all the sequences in the database (*m* sequences in total), and reordered the matrix using a fast clustering algorithm, such as hierarchical clustering [24]. This ensures that the sequences belonging to an active outbreak are grouped together. We then used a window of size *n* × *n*, and move this window along the diagonal of the *m* × *m* matrix from the top left corner to the bottom right corner. At each position of the sliding window, we used the trained CNN model to predict the label (‘epidemic’ or ‘endemic’) for the *n* × *n* submatrix of the sliding window. This sliding window approach is similar to the well-established image identification algorithm, such as R-CNN, where a subarea/window of the entire image is sampled to identify objects of interests [25]. This approach should allow the analysis of tens of thousands of sequences very efficiently.

### The HIV-1 stochastic transmission simulator

The HIV-1 stochastic forward transmission model was adapted from [26]. It has two phases: 1) an exponential growth phase and, 2) a constant population phase (Fig 1A). The simulation began with one infected individual. Secondary infections were generated stochastically according to a predefined basic reproductive number *R*_0_ (ranging between 1.5 and 5 in our simulations). It has been shown that the transmission potential is much higher during the acute infection phase in an infected individual [27, 28]. Thus, we assumed that during the first three months the rate of new infections is 20-fold higher than the remaining infectious period. In addition, we assumed that an individual will be non-infectious when on successful antiviral treatment. The time of diagnosis and antiviral treatment is assumed to be uniformly distributed between 13 to 36 months after infection [29, 30]. We also tested the robustness of model predictions by changing the shape of the distribution.

Once the population size reaches the maximum number of infected individuals, assumed to be log-uniformly distributed between 10^3^ and 10^4^ across different simulation runs, we set the population size to be constant over time. In the constant population phase, the simulation switched from generating newly infected individuals to only replacing infected individuals who are diagnosed and treated and thus no longer infectious (Fig 1A).

The transmission history of all individuals during the simulation was recorded, which allows for the reconstruction of the transmission history/tree for the entire population or any subset thereof. Samples (with a size between 15 and 50) were taken at 3 different time points. The first time point, defined as year 0, is when the population size reaches the maximum number of infected individuals, i.e., the transition time when the population changes from exponential growth to a constant size. The genealogical relationship between the samples taken at this time point reflects populations undergoing exponential growth, and therefore, we labeled the samples as ‘epidemic’. The 2nd and 3rd time points are 2 and 10 years after the first time point. The genealogical relationship between these samples therefore reflects populations that have stopped expanding, and we labelled them as ‘endemic’.

#### Construction of pairwise distance matrices

**Fig 2:**
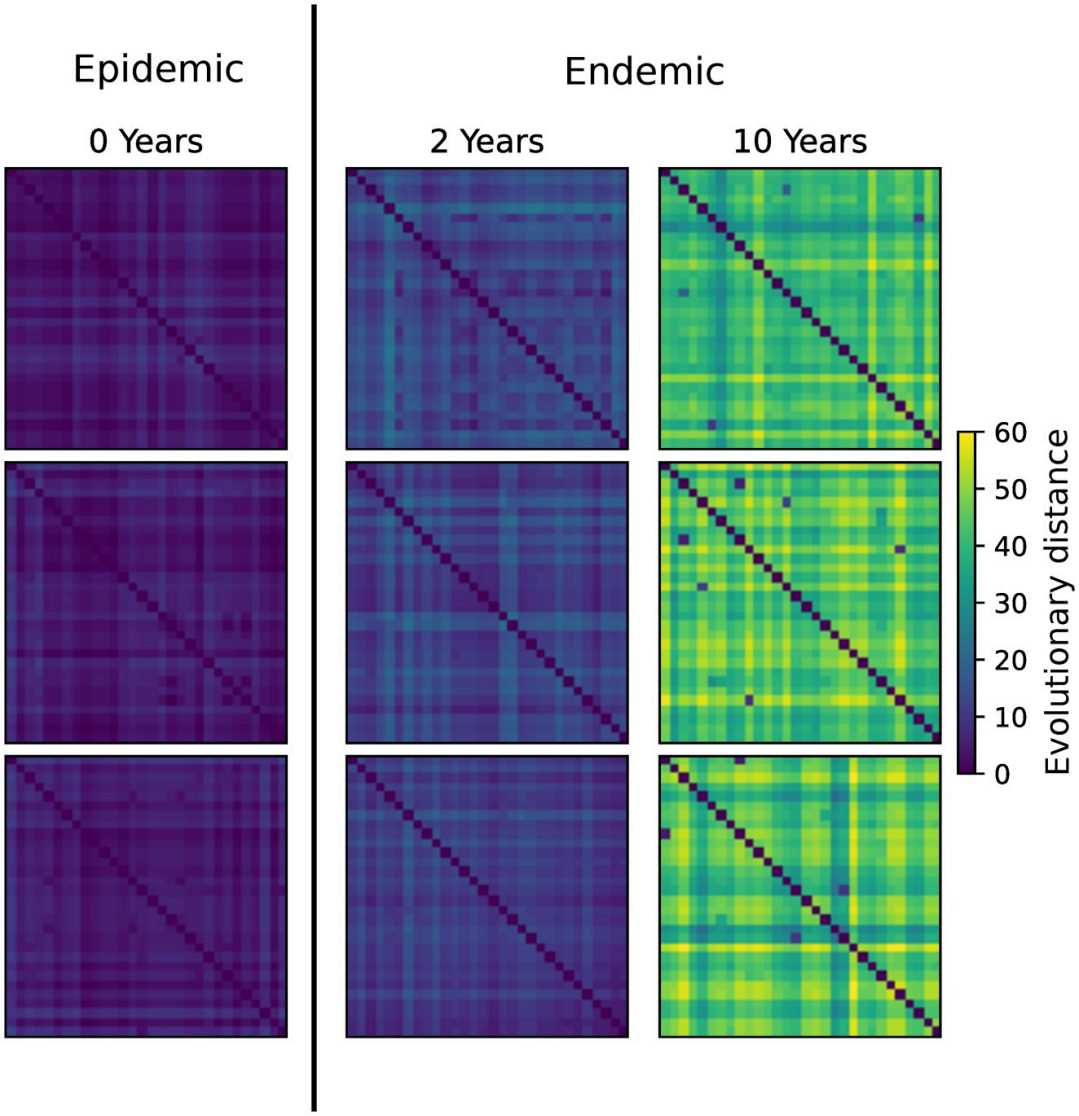
A collection of example images used for training from each sampling year. Each pictured training example was generated with *R*_0_ > 4. Three example images are shown for each sampling time (Fig 1A).

To construct an evolutionary pairwise distance matrix, *D*, we first calculated the temporal distance between each pair of samples based on the transmission history/tree. We assumed that the genetic sequence data is approximately 300 nucleotides (nt) to be consistent with the real HIV-1 data we used below. We then calculated the pairwise genetic distance separating two samples by drawing the number of substitutions from a Poisson distribution with the expected number of substitutions as the mean parameter, here 0.0067 substitutions nt^−1^ year^−1^ times 300 nt [31], times the temporal distance. This was iterated for each pair of the samples to form the pairwise distance matrix. Examples of matrices sampled from each time point (Year 0, 2, or 10) are shown in Fig 2.

### The Convolutional Neural Network (CNN) model

We developed a deep learning model using a Convolutional Neural Network (CNN) to solve a classification task predicting the label (0, 2, or 10 years) for a given pairwise distance matrix. The pairwise distance matrix is similar to a grayscale image (Fig 2). Thus, in the context of machine learning models, we also refer to a pairwise distance matrix as an image.

We constructed a CNN using Tensorflow [32] with a sequential architecture comprised of 2D convolutions, batch normalization, ReLu activations, and spatial maximum pooling. The layer structure is described in Table 1. The inputs to the first and third dense layers were regularized using dropout with probability *p* = 0.25.

**Table 1:** The layer connectivity proceeding from the input layer (top) down to the output layer. Our representation of image data within the convolutional stack is ‘channels last’. The batch size is implicitly 1 for each image and is dropped. If *k* > 20, an additional max-pool operation is performed before layer 10. No bias weight is used in the final dense layer.

Layer index	Layer type	Output shape (per image)
0	Input	(*k*, *k*, 1)
1	Convolution 2D	(*k*, *k*, 32)
2	Batch Normalization	(*k*, *k*, 32)
3	Convolution 2D	(*k* − 3, *k* − 3, 32)
4	ReLu Activation	(*k* − 3, *k* − 3, 32)
5	Convolution 2D	*k* − 5, *k* − 5, 32)
6	Max-Pool	$\left(\left\lceil \frac{k-5}{2}\right\rceil ,\left\lceil \frac{k-5}{2}\right\rceil ,32\right)$
7	Batch Normalization	$\left(\left\lceil \frac{k-5}{2}\right\rceil ,\left\lceil \frac{k-5}{2}\right\rceil ,32\right)$
8	ReLu Activation	$\left(\left\lceil \frac{k-5}{2}\right\rceil ,\left\lceil \frac{k-5}{2}\right\rceil ,32\right)$
9	Convolution 2D	$\left(\left\lceil \frac{k-5}{2}\right\rceil -2,\left\lceil \frac{k-5}{2}\right\rceil -2,32\right)$
10	Flatten	$\left({\left(\left\lceil \frac{k-5}{2}\right\rceil -2\right)}^{2}\cdot 32,1\right)$
11	Dropout	$\left({\left(\left\lceil \frac{k-5}{2}\right\rceil -2\right)}^{2}\cdot 32,1\right)$
12	Dense	(64, 1)
13	Dense	(64, 1)
14	Dropout	(64, 1)
15	Dense	(3, 1)
16	Softmax	(3, 1)

We generated five variants of this model architecture to accept different input square matrices with side lengths of 15, 20, 30, 40, or 50 (corresponding to the number of sampled individuals in the pairwise distance matrix). We refer to this parameter as the input shape or the window size interchangeably. The number and size of all convolution kernels, size of max-pool filters, and dense layer output neurons were held constant across all models. Model architecture specifications are reported in Table 1. Five batches of synthetic training data of 60,000 pairwise distance matrices were generated for each number of sampled individuals (for a total of 25 training sets), each with 20,000 examples for each label. Each dataset was used independently to train a single neural network. Each neural network was trained within under an hour using two P100 GPUs with two Power8NVL CPUs. Five validation sets of 30,000 pairwise distance matrices (10,000 matrices per label) was used to compute and compare model performance, one set for each input size. Each model was trained with a batch size of 32 images using the Adam optimizer [33] for 100 epochs with a learning rate of 10^−4^. The first and second dense layers were regularized with an *ℓ*_2_ penalty with weights of 0.05 and 0.01 respectively. Models were evaluated independently at training time and as an ensemble in deployment using a majority voting system.

### The sliding-window approach

To handle a large number of sequences, we employed a sliding-window approach. We first constructed a pairwise distance matrix *D* of size *m* × *m* for sequences in a large database. We then used a window of size *n* × *n* and moved this window from the top left corner of the diagonal to the bottom right corner of the diagonal of *D*. This method evaluates one *n* × *n* principal submatrix block in the sliding window at a time by assigning a label to the submatrix using the CNN model. For each individual represented in the pairwise distance matrix, we collected a list of the labels provided by the CNN model for each block they are represented in. The most frequently identified label within the list of predictions was assigned to an individual.

To validate the performance of the sliding window approach, we generated pairwise distance matrices of dimension 1000 × 1000 using our stochastic simulator. To generate each pairwise distance matrix, we performed 10 independent simulations and joined the resulting matrices. Each simulation generated a cluster of 100 individuals, with half of the clusters being sampled at Year 0 of the simulation, i.e. labeled ‘epidemic’, and the other half sampled at Year 2 or 10, labeled ‘endemic’. We then joined these 10 clusters into a single matrix. To ensure the 10 simulations represent distinct outbreak scenarios in the matrix, we assumed that the genetic distance between the initial infections of all 10 clusters was 15 mutations. The order of individuals in the pairwise distance matrix was then randomized. A total of 60 independent 1000 × 1000 pairwise distance matrices were generated with this process to form a validation set. Accuracy was computed on a per-individual basis using the sliding-window method.

### Robustness of model prediction against reordering and non-random sampling

We tested the robustness of model predictions against slight reordering of elements of an image using a permutation test. We asked the question: what is the probability that randomly reordering *k* individuals represented in a pairwise distance matrix results in an incorrect model prediction? To generate reordered images, we first selected *k* elements (corresponding to *k* sequences and *k* = 2, 3, or 4) in a matrix and then reordered the *k* elements through permutations. We randomly sampled with replacement 1000 possible reorderings for each image in a subset of 1000 images that were sampled from each validation set, and calculated the probability that random reordering results in the same model prediction.

As sampling effort typically follows active infections, we also tested the robustness of model predictions against non-random sampling by considering outbreaks where detection efforts are focused around a cluster of epidemiologically closely-related individuals (e.g. arising from contact tracing). To do this, we sampled a large portion of infected individuals (10%, 30%, or 50%) at the end of a simulation run. We then randomly selected a single individual and collected the closest 99 sampled infections (determined by the recorded evolutionary distance) in our larger sample. This group of 100 sub-sampled individuals is collected and represented in a pairwise distance matrix. We then joined matrices generated under this approach to obtain 60 pairwise distance matrices, and tested our model on this multiple-cluster data using the sliding window approach. We repeat this procedure at each level of sampling intensity (i.e. 10%, 30%, or 50%).

### Predicting outbreak sequences using HIV-TRACE

HIV-TRACE identifies clusters of sequences using a distance threshold [12]. These clusters are often categorized as belonging to an active outbreak. We used the simulated multiple cluster data (where each simulated cluster has 100 individuals) as described above to compare the performance of HIV-TRACE [12] with our model in identifying sequence clusters (and thus sequences belonging to outbreaks). We first normalized the simulated number of mutations by the sequence length (into substitutions/site), and set the distance threshold in HIV-TRACE at 0.015 substitutions/site. Other threshold values can give other clusters, typically higher threshold values give fewer clusters [12], a more detailed fitting procedure may be appropriate for some study populations [34]. HIV-TRACE provided a list of clusters of varying sizes. The choice of the minimal cluster size to categorize sequences belonging to an outbreak is arbitrary in general. Here we used different minimal cluster sizes, 2, 10, 25, 50, or 75 sequences (out of outbreaks with 100 sequences), and computed the sensitivity (i.e. correctly identify sequences belonging to an outbreak) and the specificity (i.e. correctly identify sequences that does not belong to an outbreak) for predictions of both the HIV-TRACE and our approach.

### Outbreak identification from real data

Applying the sliding-window approach to real HIV sequences led to predictions of sequences belonging to either an ‘epidemic’ or an ‘endemic’ phase. The prediction of a sequence collected at an earlier time may change when newly sampled sequences are added to the database, and this change may indicate an epidemiological link between an older sequence and newly sampled sequences. Therefore, for the sequences labeled as ‘epidemic’, we further considered three possible, inferred epidemiological situations based on the time a sequence was sampled relative to the time of the most recent samples (e.g. the current year).

**Fig 3:**
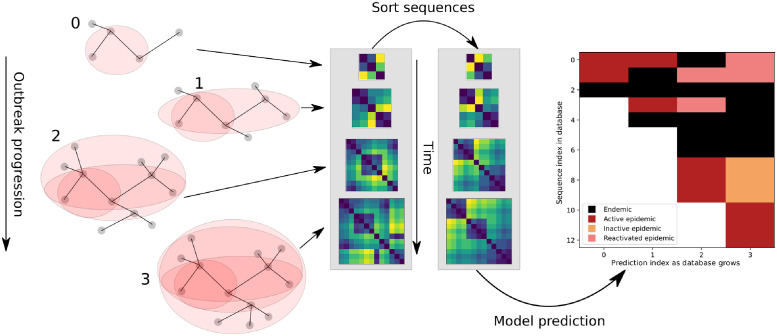
Cartoon representation of the evaluation of HIV-1 genetic data in a database in real time. As the outbreak progresses and more sequences are collected (left), sequences are added to the database. Nodes in shaded areas represent infected individuals from whom HIV-1 sequences are sampled at each time in a growing transmission network. At each time point, we construct a pairwise distance representation of the sampled sequence data and apply a sorting algorithm (middle). Collecting the predictions from each matrix gives a representation of the history and progression of an outbreak (right).

For sequences sampled within 2 years of the current year, they were labeled as part of an ‘active epidemic’. For sequences sampled more than 2 years before the current year, we categorized them according to whether they were in the same sliding window as (i.e. close to) any sequences sampled in the current year that are labeled as ‘epidemic’. If so, we labeled these sequences ‘reactivated epidemic’; otherwise, we labeled them ‘inactive epidemic’. ‘Reactivated epidemic’ indicates a situation where the newly identified outbreak may be linked to previously sampled sequences (i.e. individuals) in the database. ‘Inactive epidemic’ indicates that we predict that the sequence used to belong to an outbreak at the time when the sequence is added to the database; however, it may not be relevant to current outbreaks. The overall procedures of model prediction on an expanding database is represented in Fig 3.

### HIV sequence data used in the study

To test our model performance in a realistic setting, where sequence data enters a public health database daily over long time, we compiled data on the HIV-1 CRF01 spread in Europe. The data was sorted on sampling year, and randomized within each year. This data contains a well-known dual outbreak of HIV among first Finnish, then Swedish, intravenous drug users [35]. The sequences from that dual outbreak thus become mixed with other sequences from Europe. We arranged this database to mimic the inflow of data from an outbreak that emerges in only part of the whole data as it enters the analysis stream, one new sequence at a time. In total, this data consisted of 277 sequences covering approximately 300 nt surrounding the HIV-1 env V3 region.

To determine the sensitivity of our method to the choice of evolutionary distance model used to calculate pairwise evolutionary distances, we analyzed HIV-1 subtype A6 sequence data from Former Soviet Union, Russia, and Ukraine (417 sequences covering approximately 400 nt surrounding the env V3 region. This dataset was sampled over a 20+ year range and features long distances where a correction factor may be significant. We computed pairwise distance matrices using a Hamming model (“Ham” which counts only mutations per site), JC69, K80, F81, and TN93 using the R package ape [36, 37]. Before evaluating the model, we subset the data by year to obtain pairwise distance matrices containing sequences sampled around the same time. Years with less than the minimum number of sequences needed to make a prediction were joined forward to the next year. The resulting pairwise distance matrices were evaluated using the sliding window method and accuracy values were calculated on a per-person basis.

To test scalability, we extracted HIV-1 RT (p51) gene sequences of at least 500 nt of any subtype or recombinant in the LANL HIV database (https://www.hiv.lanl.gov/content/sequence/HIV/mainpage.html). We then removed all sequences labelled as “problematic” in the LANL HIV database and further all that required gaps, as RT typically does not have many gaps, resulting in a 1,320 nt alignment of 271,868 sequences. To estimate the runtime of our pipeline, we subsequently resampled this alignment for 10^2^ − 10^5^ sequences and constructed the pairwise distance matrix using the TN93 evolutionary model. We also measured the time to sort each matrix using hierarchical clustering and the time to stride the larger matrix into smaller views and pass the data through the model.

All HIV datasets were aligned using MAFFT version 7 [38].

## Results

### The Convolutional Neural Network (CNN) model classifies synthetic data with high accuracy

We developed a framework employing CNN models to identify active outbreaks from HIV-1 sequence data (see Methods for the workflow and details of the framework). We first generated synthetic datasets for training the CNN models using a forward stochastic simulator of HIV transmission (adapted from [26]) for training and testing of the CNN model. We varied the reproductive number, *R*_0_, between 1.5 and 5 in the simulator. Samples were taken at three different time points, i.e., during an ongoing outbreak, at year 2 or 10 after the infected population growth had ended (Fig 1A and Methods), corresponding to label 0, 1 and 2, respectively, in the CNN model. At each time point, a total of 15, 20, 30, 40, or 50 samples are taken. The sample size here is used as the sliding window size later in the general framework. For each sample size, we repeated the stochastic simulation to generate 300,000 pairwise distance matrices. The memory requirements to store the full training set for the larger sample sizes contiguously in GPU memory exceeds the capacity of many consumer-grade laptops and desktops. Therefore, we split them into five subsets of matrices with equal size, i.e., 60,000 labeled pairwise distance matrices.

**Fig 4:**
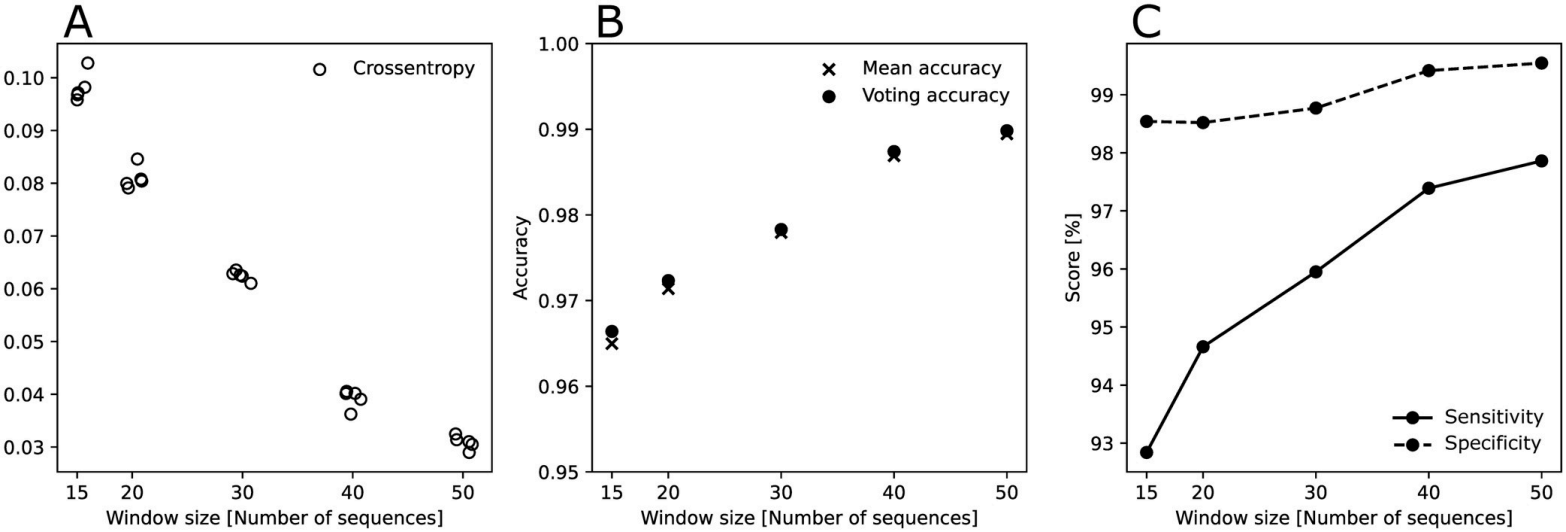
(A) Crossentropy of each trained neural network on the test sets. Crossentropy is a statistical measure of the average probability assigned to the incorrect labels by each model. Lower crossentropy indicates better model performance. Performance of networks trained on each subset of data are similar. (B) Accuracy of trained models. Constructing a voting classifier model from individual neural networks improves accuracy slightly. (C) Sensitivity (true positive rate) and specificity (true negative rate) of the voting models on the binary problem of identifying epidemic from endemic samples. Both sensitivity and specificity improve with a larger window size.

We then trained a CNN model on each of the five window size subsets of data to correctly predict the labels (i.e. label 0, 1, and 2) of the pairwise matrices. Similar model performance were achieved across the five subsets (Fig 4A). We then joined all the trained networks in a simple bagging classifier with a voting decision method. This led to a classifier with a higher accuracy than the mean accuracy of the five networks used to assemble the larger classifier model (Fig 4B). We thus used the voting classifier for predictions below. Overall, the accuracy of the CNN model with the voting decision method ranged between 93% and 99%. The accuracy improved with increased sample size (i.e., larger windows perform better; Fig 4).

From a public health perspective, we are interested in identifying sequences in a outbreak (i.e. label 0 in our CNN model) among sequences collected from an endemic (non-outbreak; labels 1 and 2 in our CNN model) phase. We thus classified label 0 as ‘epidemic’, and labels 1 and 2 as ‘endemic’. We found that at all window sizes, the sensitivity (i.e. correctly identifying ‘epidemic’ sequences) exceeds 92%, and the specificity (i.e. correctly identifying ‘endemic’ sequences) exceeds 98%. Sensitivity increases notably with increases of the window size.

### The convolutional neural network based model performs better when the outbreak is more explosive

**Fig 5:**
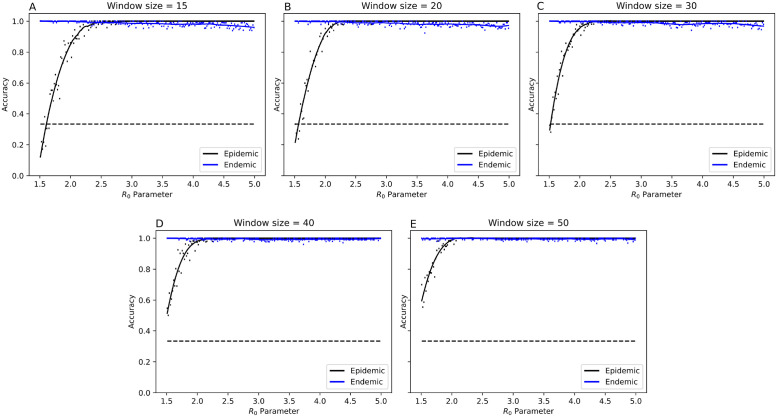
Association between *R*_0_ parameter and per-image model performance by epidemic vs endemic training label. The *R*_0_ axis is subdivided into 200 subintervals. Accuracy is calculated over each subinterval, a trend line is fitted using a Savitzky-Golay filter of degree 3 with a window size of 51. A black dashed line indicates the probability of predicting an outbreak using only the occurrence rate in the training set, independent of *R*_0_. Outbreaks with small *R*_0_ are more difficult to detect than with larger *R*_0_.

The basic reproductive number *R*_0_ is one of the key parameters characterizing an outbreak. We analyzed the performance of our model in relation to the *R*_0_ parameter used during simulation. Matrices in the training set were partitioned by label (‘epidemic’ or ‘endemic’), and then binned according to the value of *R*_0_ into 200 uniformly sized bins. Fig 5 shows the average accuracy for each bin. Our model performed well at identifying sequences taken from the endemic phases. In terms of identifying sequences sampled from an epidemic, the model accuracy exceeded 90%, when *R*_0_ > 2.5. Epidemics with *R*_0_ less than 2.5 were difficult to detect. However, increasing the sample size increased the probability of identifying an outbreak notably, decreasing the threshold for high accuracy to *R*_0_ > 2 with a window size of 50.

In addition, we further evaluated how the maximum population size in the simulator impacts the accuracy of model prediction (S1 Fig). In general, the model performance is less impacted by the choice of this parameter.

### The sliding window approach identifies subsets of sequences taken from outbreaks

Typically, the number of sequences in a database is much larger than 50. To handle the large number sequences, we used a sliding window approach (see Methods). In this approach, we first constructed a pairwise distance matrix for all the sequences in the database, and sort the matrix so that closely related sequences are grouped together. We then employed a sliding window (of size 15, 20, 30, 40, or 50) and move this window along the diagonal of the matrix. We make predictions to the group of sequences in the window at each window position using the CNN model. This leads to a list of prediction labels for each individual while the window moves. The most frequently identified label within the list of predictions was assigned to the individual (see Methods for details).

**Fig 6:**
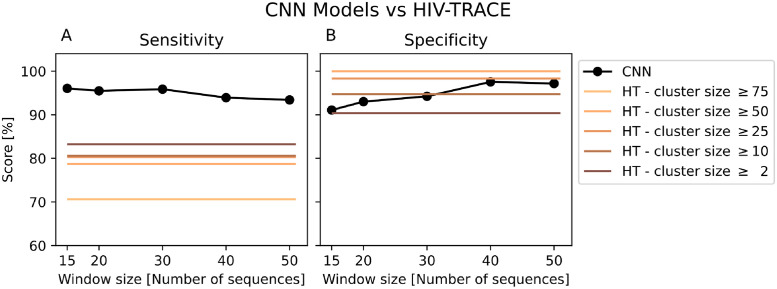
Sensitivity and specificity on the multiple-cluster test set of our method and HIV-TRACE. Model performance is computed using the same 60 matrices of 10 clusters each for a total of 60, 000 infections. (A) Sensitivity is high and exceeds all interpretations of HIV-TRACE output. (B) Specificity of the method increases as the window size increases and performs comparably to HIV-TRACE.

For sorting the pairwise matrix for sequences in the database, We tested three different methods: 1) randomly ordered, 2) hierarchical clustering (Ward criterion), and 3) hierarchical clustering (Ward criterion) with optimal leaf ordering refinement. To test the performance of the three methods, we generated 60 pairwise matrices (images) of size 1000 by 1000. Each of the 1000 by 1000 matrices was generated by joining 10 simulated matrices using the stochastic simulator with outbreak parameters (*R*_0_ and simulation length) randomly sampled (see Methods for details). Half of the sequences were sampled at year 0 (i.e. ‘epidemic’) and the other half were sampled in year 2 or 10. In total, we evaluated 60,000 predictions to compute the accuracy of the models using the per-person prediction rule to interpret their outputs. Of the three tested sorting methods, applying hierarchical clustering gave the best accuracy (see S2 Fig). Thus, hereafter, we applied this sorting method to all large matrices. Overall, applying our approach to this multi-cluster dataset, we obtained a sensitivity greater than 93% across different window size used (Fig 6). The specificity of our approach increased from 91% to above 95% when the window size increases.

### Comparison of performance with HIV-TRACE

A popular tool for analyzing sequence data using a distance based approach is HIV-TRACE [12]: a pipeline for processing sequence data using pairwise distances and distance thresholds. It aims to detect transmission clusters by examining individual distances. Usually, sequences belonging to clusters of large sizes are categorized as sequences belonging to an outbreak. Here, we compared the performance of our approach to HIV-TRACE in terms of identifying sequences sampled from outbreaks. To deploy HIV-TRACE, the distance threshold for determining outbreak clusters must be determined a priori. Here we use the suggested distance threshold of 0.015 substitutions/site [12]. We considered various different cluster size thresholds, i.e. 2, 10, 25, 50, or 75, above which the clusters are categorized as belonging to outbreaks. See Methods for details.

Overall, our approach performed much better than HIV-TRACE in detecting sequences belonging to an outbreak (Fig 6). For example, irrespective of the cluster size threshold used in HIV-TRACE, the sensitivity is below 85% (compared to greater than 93% for our model). For HIV-TRACE, increasing the cluster size threshold achieved greater specificity, with the cost of lower sensitivity to identify sequences belonging to outbreaks.

### Robustness analyses

We tested the robustness of our model performance against variations in a variety of parameters we assumed in the model. First, in our model, we assumed that the time when a person is diagnosed and thus becomes non-infectious follows a uniform distribution (between 13 and 36 months). Here we tested the impact of using an exponential model for time until diagnosis and treatment by repeating the analysis with data generated under this alternative assumption. We found that our predictions is robust to variation of this assumption (S3 Fig).

Our model used a fast clustering algorithm, i.e. hierarchical clustering, to group closely related sequences into clusters. The clustering algorithm is not as thorough as a proper phylogenetic search, nor does it have to be; it only needs to order sequences approximately around similar sequences. We thus tested whether the ordering of the elements or sequences in a pairwise distance matrix (within a sliding window) matters for model prediction. We permuted two, three, or four randomly chosen sequences in a matrix and calculate the probability that the model gives the correct prediction (Table A in S1 Text). Note that reordering of the sequences could lead to a different image (pairwise-distance matrix) on which the CNN model makes its prediction. Overall, a random reordering of up to four sequences gives a correct prediction with a probability of over 96% and this percentage remains relatively constant as *n* increases. Thus, our method is robust to the specific ordering of the sequences presented to the model in a sliding window. This suggests that the precise evolutionary relationship between sequences is not necessary for our CNN model to make correct predictions as long as closely related sequences are grouped together (through clustering algorithms, which typically are faster than phylogenetic reconstruction).

Third, as sampling and sequencing effort is often structured rather than random, we tested our model on large matrices obtained using a cluster-driven sampling technique (see Methods). Each cluster is obtained by specifying a sampling intensity (10%, 30%, or 50%) which makes a proportion of the simulated population available, then selecting the closest available neighbors to a randomly selected infection, to obtain a sample. With a sampling intensity of 10% (10% of the population is available for inclusion in the sample), similar quality predictions to random sampling are obtained (Fig 6A and 6B, S4 Fig. Increasing the sampling intensity beyond 10% leads to a decrease in specificity, while sensitivity attains its maximum possible value at 100% (S4 Fig). Overall, this method is robust to some non-randomness in the sampling.

Fourth, we tested the robustness of model predictions using different evolutionary models to compute pairwise evolutionary distances from HIV-1 sequence data. We used a meta-dataset of HIV-1 sequences from the former Soviet Union [39] (see Methods). Overall, for models using all window sizes (15, 20, 30, 40, or 50), there is minimal pairwise variation between the predictions using different evolutionary models (S5 Fig).

### Validating model using historical HIV-1 data

**Fig 7:**
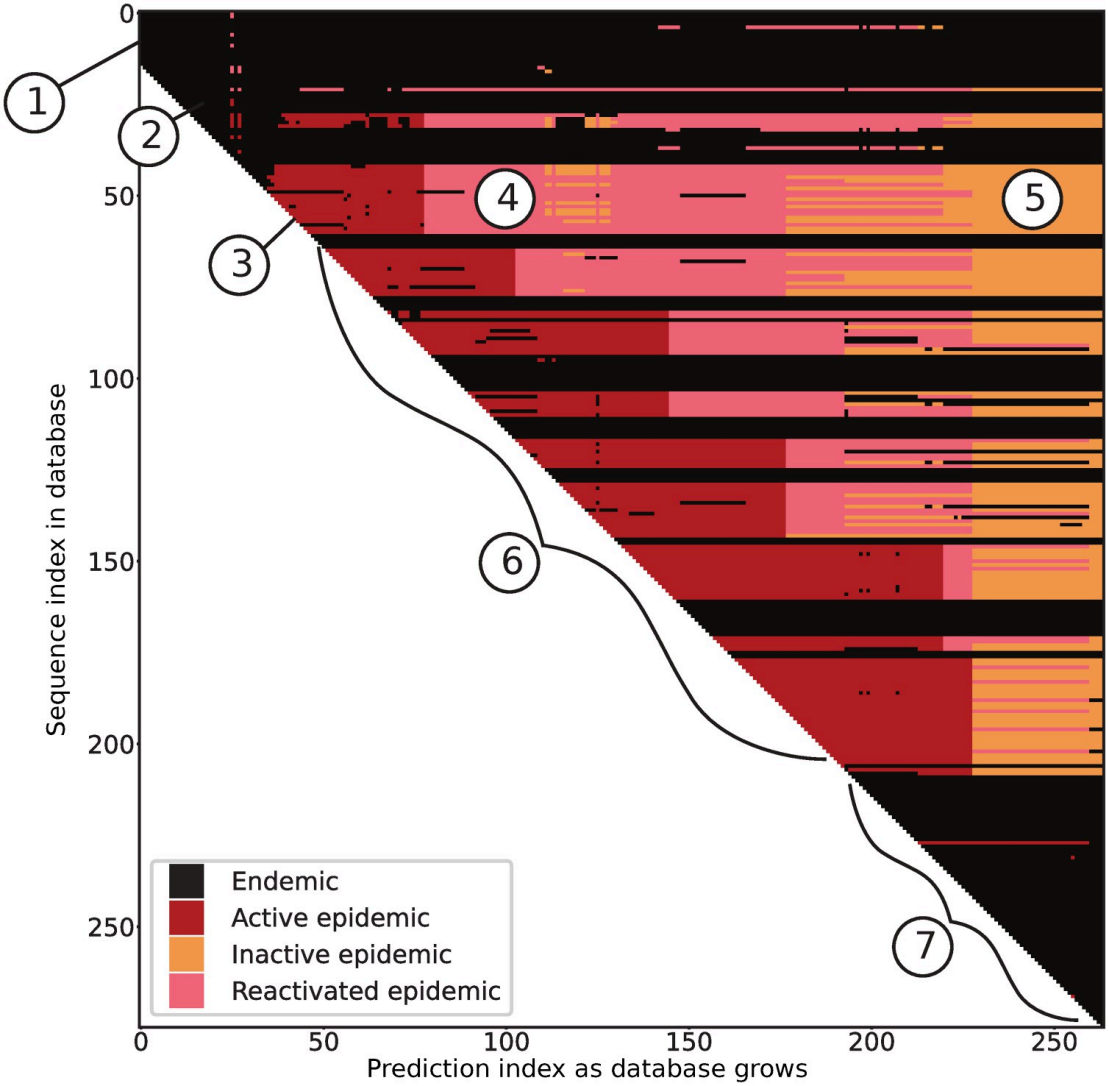
Real-time detection of a HIV-1 CRF01 outbreak (see Fig 3 for methodology). As sequences are added to a growing database (from top to bottom on the y-axis) and time progresses (x-axis), one distance matrix is analyzed at each sequence addition and an updated classification of all current sequences is added as a slice along the x-axis. Our model classifies each infection as belonging to an outbreak (epidemic) or as endemic. The epidemic label is refined into active, inactive, or reactivated based on the time since detection. Here, the transition cutoff from active epidemic to inactive/reactivated epidemic is 2 years. Circled numbers indicate events of interest, discussed in the main text.

To validate our model against real datasets and illustrate the application of a real outbreak identification in real time, we collected a dataset containing a well-documented dual outbreak among first Finnish and then Swedish IDUs [35]. To add complexity, we added an assortment of other European HIV-1 infections from the time period around the Finnish-Swedish outbreak to test our model’s ability to detect an outbreak in an endemic backdrop (Fig 7). An initial subset of 15 sequences is required to make a first prediction (‘circled 1’ in Fig 7). These initial 15 sequences were classified as endemic, which agreed with how they were sampled; coming from different European countries (including Sweden), not as part of any known outbreak. Note that most of these initially classified endemic sequences stayed endemic throughout the growth of the database.

After the initial 15 sequences, the database grows one sequence at a time, and we calculated a new distance matrix for all sequences up to that point, applied sorting, and performed predictions using the sliding window as outlined in Fig 3. The sequences were re-classified in each matrix step, and the result added as a slice along the x-axis. At ‘circled 2’, additional sequences were added as endemic, but shortly thereafter, when sequences were added at ‘circled 3’, their classification changed to ‘active epidemic’ as new sequences inform about an ongoing outbreak. This correctly identified the start of the Finnish IDU outbreak in 1999. Sequences that joined the database at ‘circled 3’ and were classified as active epidemic, later became classified as ‘reactivated epidemic’ at ‘circled 4’ as they were about to become ‘inactive epidemic’ but new active epidemic sequences were added, instantly reactivating them. This suggests that sequences in ‘circled 4’ were linked with newly added sequences in ‘circled 6’. At ‘circled 5’, these (and many other) sequences eventually became inactive epidemic state.

During ‘circled 6’, first, the Finnish outbreak developed as shown in red; interspersed with unrelated sequences from patients in Denmark and the UK added correctly in black; then, while Finnish sequences kept being added in red, some Swedish IDU sequences were added in black around year 2001, these were correctly identifying pre-outbreak slow spread in Sweden [35], again with various sequences from other European countries that were not part of any known outbreak also added in black. Eventually, in 2004, the well-documented Swedish IDU outbreak started, accurately identified by our model.

At the end of the database growth, around ‘circled 7’, the final 69 sequence additions (except one) joined as endemic state (black). This involved both Finnish and Swedish post-outbreak slower spreading HIV in the IDU group, as well as other European sequences from the same time period. At the final slice in Fig 3, all sequences were classified as either ‘endemic’ or ‘inactive epidemic’, correctly indicating no new outbreak in these data at that time.

Overall, our model correctly identified the epidemiological dynamics of the Finnish-Swedish IDU outbreaks among other European HIV-1 CRF01 sequences during the same time period as the outbreaks happened. Importantly, we detected both outbreaks early on, before they were over, implying that had this method been applied in real-time as data became available, we would have been able to intervene and possibly prevent the extent of the outbreaks.

### Our approach efficiently handles over 100,000 sequences

**Fig 8:**
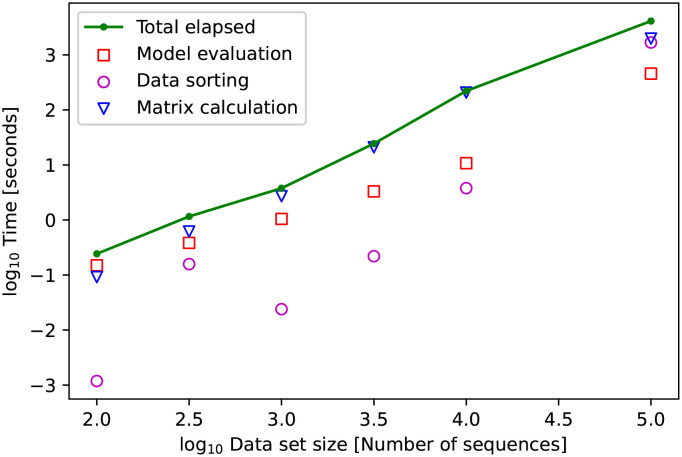
Decomposition of measured total method runtime into the primary components of distance matrix construction, distance matrix sorting, and model evaluation. Measured time performance averaged then log transformed. Less than 100 sequences can be analyzed in under 1 second.

Finally, we evaluated the ability of our model to perform under increasing quantities of sequences. We downloaded 271,868 aligned HIV-1 pol sequences from the LANL HIV sequence database and subsampled sets ranging from 10^2^ sequences to 10^5^ sequences (see Methods). We measured the real time it took to construct a large pairwise distance matrix from the sample using ape [36], the time required to sort the matrix using hierarchical clustering [40], and the time required slide the window down the sorted matrix diagonal and pass the data through the model to identify sequences belonging to an epidemic. Measured times were averaged at each data set size then log transformed and plotted in Fig 8. A set of 100,000 sequences were analyzed with this pipeline in under 70 minutes. Note that we did not consider the time to run the simulator to generate the synthetic data and train the CNN model, because these tasks can be performed offline (before outbreak analysis).

In addition to the empirical results, we consider the computational complexity of these operations as the number of sequences in the dataset, *n*, increases. The sliding window method for annotating large matrices has *O*(*n*) time complexity as the number of model evaluations and the complexity of such evaluations grows linearly with the number of infections presented. Constructing the pairwise distance matrix has *O*(*n*^2^) time complexity, and hierarchical clustering implemented with nearest neighbor chaining is performed with *O*(*n*^2^) time complexity [24] giving a time complexity approximately *O*(*n*^2^) for the full pipeline.

## Discussion

In this work, we developed a deep learning approach to effectively and efficiently handle large quantities of viral sequences to identify subsets of sequences that were taken from an HIV-1 active outbreak. Our approach constructs and directly analyzes pairwise distance matrices from viral sequences. The key idea is to treat these matrices as images and then train convolutional neural network (CNN) models to identify subsets of sequences which form matrices that bear the signature of an outbreak. Using historical HIV-1 genetic sequence data with known epidemiological history, we showed that our approach correctly identified subsets of sequences sampled from outbreaks. Furthermore, we showed that our approach is scalable and can process hundreds of thousands of sequences within hours.

The key to the low-computational cost of our approach is analyzing a pairwise distance matrix derived from sequences in a database, instead of constructing phylogenetic trees. While computing large trees from the full sequences is possible using fast, but approximate, methods, subsequent interpretation is ad hoc, often subjective, and requires careful analysis by experts. Other, more advanced methods that partially incorporate interpretation and integrating across many plausible trees, such as elegant Bayesian methods [11], are instead slower and unsuitable for very large data analyses. On the other hand, while the information encoded in a pairwise distance matrix is significantly less than that encoded in the full sequences, it has been shown that this dimension reduction does not substantially impact the information content with respect to the evolutionary relationships between sequences [41]. By building the pairwise distance matrix, we concentrated the epidemiological signatures (outbreak or endemic) into a more compact structure, i.e. an image format, that can be analyzed very efficiently using deep learning. Given the trained model, the computational speed and complexity of our approach is currently limited by the quadratic complexity (in the number of sequences considered) in building the pairwise distance matrix and secondarily by the data sorting and model evaluation.

The accuracy of the model prediction is ensured by applying a deep learning method based on CNN models for image classification. The model was trained using a large set of synthetic data, i.e. pairwise distance matrices with various sizes (defined as window sizes), generated from a previously validated HIV-1 transmission simulator [26]. Our method had better specificity with larger window sizes, but the obvious downside of larger windows is that we need larger number sequences to make a prediction. Thus, for HIV-1, we recommend to keep the window size at 15 sequences, while for other viruses where a large amount of sequence data are made available each day, such as SARS-CoV-2, a larger window may perform better.

Our deep learning model performed well when *R*_0_ is high (*R*_0_ > 2.5). Large *R*_0_ characterizes fast-growing outbreaks that we would like to identify early and with high confidence. When *R*_0_ is small, the outbreak grows significantly slower and is difficult for our model to detect. Further, when the mutation rate is lower than what we assumed in our model, the signal from genetic distances would become weaker, and thus the performance of our outbreak prediction model would decrease. More sophisticated CNN models than proposed here may be used in future improvements to the ability of the model to detect outbreaks. However, another factor that may lead to poor model performance is lack of signal in the data. For example, increasing the sample size from 15 sequences to 30 or 50 sequences does not give a substantial increase in performance for low *R*_0_ (S1 Fig). This further suggests lack of signal for detection.

The assumption of having independent random samples from the same outbreak may be unrealistic in some instances. We tested model robustness against this assumption by sampling a large fraction of individuals (10%, 30%, or 50%) from the infected population to emulate increased sampling intensity around an initial infection discovered by surveillance efforts. Our results showed some robustness to this alternative sampling scheme, but model accuracy decreases as the sequencing effort increases above 10% (so more than 10% of the entire outbreak is sequenced). This led to closely related sequences being sampled giving pairwise distance matrices that are more similar to ‘epidemic’ labeled pairwise distance matrices than the correct ‘endemic’ label. To effectively handle the issue of non-random sampling, further work is needed to train the CNN model with data generated from a model assuming varying sampling intensities.

Because our method is based on analysing a distance matrix, it is interesting to compare it to HIV-TRACE, i.e. a distance matrix-based approach [12]. One potential issue with the distance threshold in HIV-TRACE is that it often broke up linked outbreak sequences into smaller separate clusters. In contrast, our method does not assign sequences to specific clusters, it simply assigns sequences to outbreak or not. Thus, our method and HIV-TRACE provide somewhat different types of results related to outbreak identification. We accommodated this difference by using different minimum cluster sizes in HIV-TRACE for categorizing sequences as belonging to an outbreak. On simulated data, our method performed much better in sensitivity while it was similar in specificity (Fig 6).The performances of both our current model and HIV-TRACE suffer from non-random sampling of infected individuals.

Overall, our deep learning model is capable of processing tens of thousands of sequences, and making reliable predictions on whether a sequence is taken from an outbreak. We validated this model using a set of real HIV-1 sequence data, and showed that the model detected a known dual outbreak among injecting drug users first in Finland and then in Sweden, intermingled by random data from the same subtype in Europe, accurately identified the outbreak in a data stream where HIV sequences were provided over the time the outbreaks occurred. Encouragingly, the model detected the outbreaks early, before they were over, as well as the end of the outbreaks. Accurately identifying a beginning outbreak in the background of other data and knowing when it is over are both crucial pieces of information for a public health authority. More broadly, while we developed this computational framework for HIV-1, with adjustments in the evolutionary rate and training simulations, it could be applied to analyze other pathogens for online, rapid response to developing outbreaks and rising of novel variants of concerns, e.g. for SARS-CoV-2.

## Supporting information

S1 Fig Analysis of accuracy as a function of both population size (*N*_*pop*_) and *R*_0_. Each dimension is binned into 30 subintervals. Accuracy is calculated on 10,000 sample images for each combination of outbreak status and window size.
(EPS)

S2 Fig Performance of the sliding window algorithm with different clustering methods applied to the full data set. Individuals represented in each cluster are randomly sampled from the simulated population. Half of the individuals are drawn from an outbreak, the remaining half are from endemic scenarios. Method HC denotes hierarchical clustering, OLO denotes HC with optimal leaf ordering refinement, None denotes no sorting is applied. Accuracy is computed using the three labels used during training.
(EPS)

S3 Fig Cross-validation of data/models on validation sets with exponentially distributed time until treatment/diagnosis (still with a mean duration of 2 years) vs the uniform assumption considered in Fig 5. Model accuracy is largely unchanged when trained or evaluated on data generated under the exponential duration assumption vs. the uniform assumption. Sensitivity to low *R*_0_ is robust to this variation.
(EPS)

S4 Fig Comparison of CNN models with HIV-TRACE on cluster-sampled data. Sensitivity (A,C,E) and Specificity (B,D,F) of CNN models against HIV-TRACE with varying filter rules. Each row denotes a different sampling intensity (10%, 30%, or 50%) which corresponds to the total proportion of the simulated population that could be included in the matrix for the simulation. Higher sampling intensities result in closer genetic relationships being represented. Sensitivity remains high while specificity declines as the proportion of false positive detection events increases.
(EPS)

S5 Fig Similarity of model predictions on Russian dataset when presented by year. Models with smaller window sizes give similar predictions, an advantage over models with larger window sizes.
(EPS)

S1 Text Supporting tables for neural network construction and model robustness against reordering. (PDF)
