# Supplementary material for: A deep learning approach to real-time HIV outbreak detection using genetic data
Source: PLoS Comput Biol. 2022 Oct 14;18(10):e1010598. doi: 10.1371/journal.pcbi.1010598 (PMC9604978; doi:10.1371/journal.pcbi.1010598)
Supplement: S1 Text — (PDF) [file pcbi.1010598.s006.pdf]

Table A: **The number of trainable parameters in models increases as the number of samples used to generate each input matrix increases.**

| Sample Size ($k$) | Trainable Parameters | Trainable Parameters in Dense Layers |
|---|---:|---:|
| 15 | 42,048 | 6,464 |
| 20 | 72,896 | 37,184 |
| 30 | 72,896 | 37,184 |
| 40 | 140,352 | 104,768 |
| 50 | 205,888 | 170,304 |

Table B: **The probability of reordering individuals within the matrix and obtaining a correct prediction is a measure of model performance against reordering.** A subset of 1000 matrices are sampled from the test set, are sorted using hierarchical clustering. For each matrix, 1000 possible reorderings of up to 4 individuals for each matrix are sampled with replacement, and then evaluated with the model.

| $w_s$ \ $n$ | 2 | 3 | 4 |
|---|---:|---:|---:|
| 15 | 0.967848 | 0.967663 | 0.967760 |
| 20 | 0.961021 | 0.960988 | 0.960925 |
| 30 | 0.961502 | 0.961561 | 0.961545 |
| 40 | 0.974899 | 0.974925 | 0.974894 |
| 50 | 0.963433 | 0.963418 | 0.963477 |

Calculated probability that a reordering of any two individuals gives the correct prediction on 1000 matrices sampled from the validation set used during training. Here $w_s$ denotes the window size and $n$ is the number of elements permuted.
